# An updated map of *Trypanosoma cruzi* histone post-translational modifications

**DOI:** 10.1038/s41597-021-00818-w

**Published:** 2021-03-25

**Authors:** Rafael Fogaça de Almeida, Matheus Fernandes, Lyris Martins Franco de Godoy

**Affiliations:** Instituto Carlos Chagas, Fiocruz Parana, Curitiba, Parana Brazil

**Keywords:** Parasitology, Proteomics

## Abstract

In humans and other eukaryotes, histone post-translational modifications (hPTMs) play an essential role in the epigenetic control of gene expression. In trypanosomatid parasites, conversely, gene regulation occurs mainly at the post-transcriptional level. However, our group has recently shown that hPTMs are abundant and varied in *Trypanosoma cruzi*, the etiological agent of Chagas Disease, signaling for possible conserved epigenetic functions. Here, we applied an optimized mass spectrometry-based proteomic workflow to provide a high-confidence comprehensive map of hPTMs, distributed in all canonical, variant and linker histones of *T. cruzi*. Our work expands the number of known *T. cruzi* hPTMs by almost 2-fold, representing the largest dataset of hPTMs available to any trypanosomatid to date, and can be used as a basis for functional studies on the dynamic regulation of chromatin by epigenetic mechanisms and the selection of candidates for the development of epigenetic drugs against trypanosomatids.

## Abstract (summary)

**Table:** 

Measurement(s)	histone_modification
Technology Type(s)	mass spectrometry • nanoflow liquid chromatography-tandem mass spectrometry • Data-Dependent Acquisition
Sample Characteristic - Organism	Trypanosoma cruzi

Machine-accessible metadata file describing the reported data: 10.6084/m9.figshare.13491165

## Background & Summary

*Trypanosoma cruzi*, the causative agent of Chagas Disease, is a flagellated protozoan belonging to the order Kinetoplastida, family Trypanosomatidae^1^. About 6 to 7 million people are estimated to be infected with *T. cruzi* worldwide^2^, making it a serious public health problem. In its life cycle, *T. cruzi* passes through different hosts, including mammals and insects and, to adapt to these different environments, undergoes dramatic changes in its phenotype, which require a fine regulation of gene expression.

The regulation of gene expression in an organism can occur in different levels. In humans and most other eukaryotes, one of the key points of gene expression control is the regulation of transcription initiation by epigenetic mechanisms, such as the occurrence of hPTMs^3^, and “epigenetic drugs” are already in use for the treatment of different diseases, such as cancer and neurological disorders^4–6^. In *T. cruzi* and other trypanosomatids, on the other hand, the control of gene expression occurs mainly post-transcriptionally, at the level of RNA and protein^7^. Despite that fact, the chromatin of *T. cruzi* is similar to that of other eukaryotes. It is organized into chromosomes containing canonical (H2A, H2B, H3 and H4), variant (H2A.Z, H2B.V and H3.V) and linker (H1) histones^8^ and the presence of common hPTMs (acetylation, methylation and phosphorylation) has been detected in both their replicative and non-replicative forms^9–19^. More recently, our group identified a plethora of 13 different hPTM types in *T. cruzi* epimastigotes, starting to unravel a histone code that potentially supports the existence of chromatin regulation via post-translational modification in this parasite^20^.

The importance of a few modification types and specific hPTM marks for trypanosomatids have been demonstrated. In *T. brucei*, di- and trimethylation of H3K76 regulate the cell cycle^21^; methylation and acetylation act as signaling for histone writers and erasers in the regulation of variant surface glycoproteins (VSGs)^22,23^ and variant histones and H3 trimethylation are enriched in probable transcription initiation sites^24,25^. Similarly, the origins of polycistronic transcription seem to be regulated by histone acetylation in *Leishmania major*^26^ and, in *T. cruzi*, acetylation and methylation indicate regions of transcriptional initiation of divergent polycistronic transcription units, which contain evolutionarily conserved bidirectional promoters^11^. However, the global impact of hPTMs, as well as the mechanisms underlying their function in trypanosomatids and the language of crosstalk regulation between different PTMs, are still poorly understood.

For trypanosomatids of medical interest, particularly for *T. cruzi*, the lack of basic information about epigenetic molecular players, such as the thorough identification and site-specific localization of histone PTMs marks, has hindered functional research that would allow the understanding of epigenetic control and the identification of targets for epigenetic drugs. Nevertheless, due to the high degree of similarity of its orthologous genes, it is very likely that epigenetic components are also essential for the growth and survival of this parasite and can represent important targets for the development of new therapies for Chagas Disease.

**Fig. 1:**
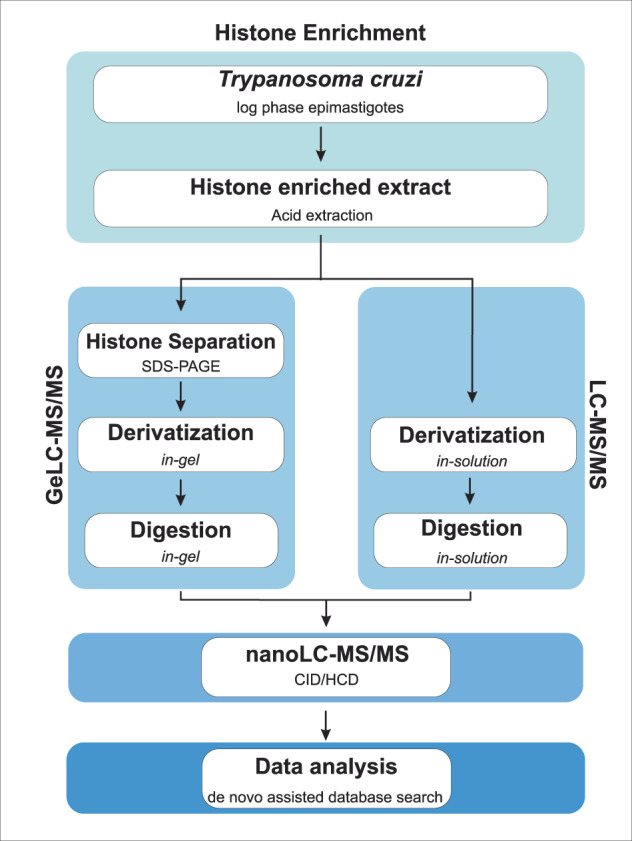
Experimental workflow for the comprehensive profiling of *T. cruzi* hPTMs.

**Fig. 2:**
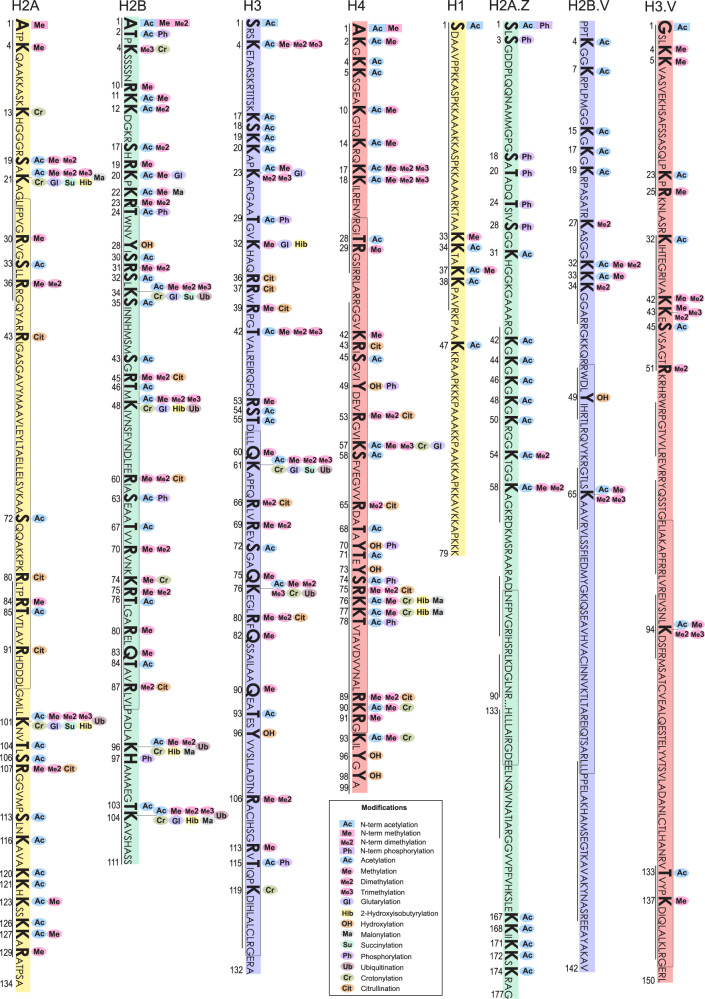
Global map of hPTMs currently described for *T. cruzi*. Modification sites are marked in bold letters and the numbers below the sequence represent the position of the amino acid in the sequence (after removal of the initial methionine). Rectangles indicate the histone-fold within the globular domain. The line below the sequence represents the region covered in our experiments.

**Fig. 3:**
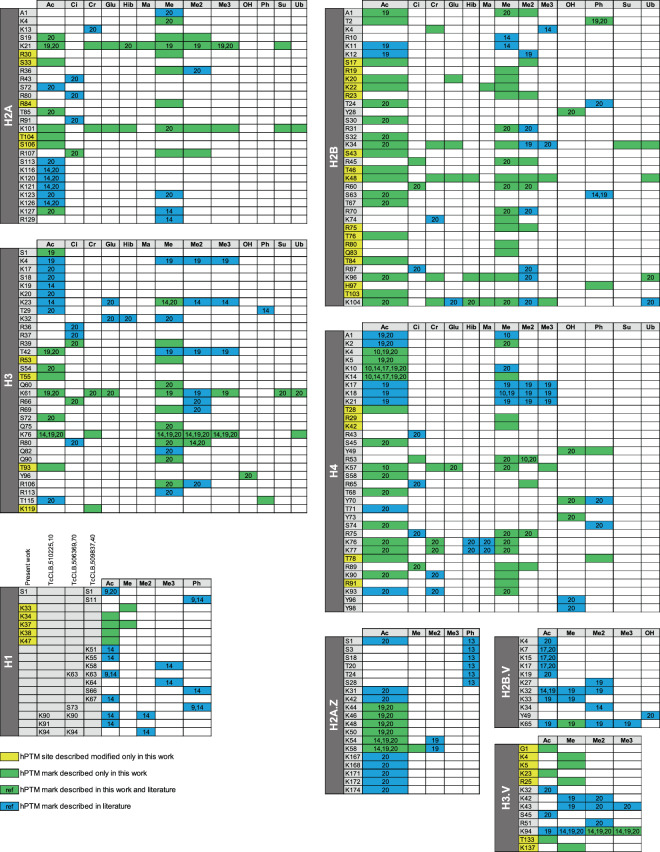
Summary of the *T. cruzi* hPTMs described in the present work and the literature. Colors indicate which hPTM sites and marks were confirmed and which ones are novel. For the confirmed ones, the reference numbers indicate the respective work(s) where each modification site or PTM mark was previously described. Ac (acetylation), Ci (citrullination), Cr (crotonylation), Glu (glutarylation), Hib (hydroxyisobutyrylation), Ma (malonylation), Me (monomethylation), Me2 (dimethylation), Me3 (trimethylation), OH (hydroxylation), Ph (phosphorylation), (K), Su (succinylation), Ub (ubiquitination).

In this context, our group has been working on large-scale proteomic analysis to provide a global view of the PTM landscape for each of the *T. cruzi* histones, aiming to smooth this gap of information and to pave the way for functional epigenetic studies on trypanosomatids. Here, we applied optimized sample preparation, two parallel mass spectrometry-based proteomic approaches (GeLC-MS/MS and LC-MS/MS) with complimentary sensitive/high-resolution fragmentation techniques (CID/HCD) and *de novo* assisted database search (Fig. 1) to deeply profile the PTMs of *T. cruzi* canonical, variant and linker histones, increasing to 189 the number of hPTM sites and to 353 the number of hPTM marks described for this parasite (Fig. 2) and contributing to the hypothesis of the existence of dynamic regulation of chromatin by hPTMs in trypanosomatids. A summary of the global numbers of *T. cruzi* hPTMs described up to this date is available in Table 1 and detailed in Fig. 3. Our updated *T. cruzi* hPTM dataset represents the most comprehensive available for any trypanosomatid to date, and can be used as a basis for future functional studies and selection of targets for the development of anti-parasitic epigenetic drugs.

**Table 1:** Global numbers for hPTM identification of *T. cruzi* histones.

	This work			Total described	
	Sequence coverage (%)	hPTM sites (confirmed/novel)	hPTM marks (confirmed/novel)	hPTM sites	hPTM marks
H2A	64	13 (8/5)	33 (11/22)	26	49
H2B	95	32 (17/15)	68 (21/47)	36	87
H3	97	21 (17/4)	34 (25/9)	32	64
H4	97	26 (21/5)	43 (28/15)	34	74
H2A.Z	34	6 (6/0)	7 (6/1)	19	23
H2B.V	38	1 (1/0)	1 (1/0)	11	17
H3.V	65	8 (1/7)	9 (2/7)	13	19
H1	27	5 (0/5)	6 (0/6)	18	20
	**Total**	112 (71/41)	201 (94/107)	189	353

## Methods

### Cell culture and histone enrichment

*T. cruzi* Dm28c epimastigotes were cultured to log phase in liver infusion tryptose (LIT) medium^27^, supplemented with 10% fetal bovine serum without agitation at 28 °C. Histone extraction and enrichment were performed as previously described^20^, with some modifications. Briefly, 1 × 10^9^ epimastigote cells were collected by centrifugation (10 minutes, 5000 g at 4 °C). Cells were lysed resuspending the obtained pellet in 1 ml of extraction buffer A (250 mM Sucrose; 1 mM EDTA; 3 mM CaCl_2_; 10 mM Tris-HCl pH 7.4; 0.5% (v/v) Saponin; 10 mM sodium butyrate, 1x protease inhibitor cocktail (Complete Mini EDTA free, Roche) and 1x phosphatase inhibitor cocktail (Roche)) and centrifuged for 10 minutes at 6000 g, 4 °C. Cell pellet was washed in 1 ml of extraction buffer B (extraction buffer A without saponin) and centrifuged for 10 minutes at 6000 g, 4 °C. The pellet, containing the cell nuclei, was resuspended in 1 ml of Buffer C (1% (v/v) Triton X-100; 150 mM NaCl; 25 mM EDTA; 10 mM Tris-HCl pH 8; 10 mM sodium butyrate, 1x protease inhibitor cocktail (Complete Mini EDTA free, Roche) and 1x phosphatase inhibitor cocktail (Roche)) and then centrifuged for 20 minutes at 12000 g, 4 °C. The pellet was washed 3 times in 100 mM Tris-HCl pH 8, resuspended in 1 ml of 0.4 N HCl and incubated on a rotator overnight at 4 °C. Acid soluble proteins were recovered in the supernatant after sample centrifugation for 15 minutes at 10000 g, 4 °C. The supernatant was transferred to a clean tube; acetone (8 x the initial volume) was added and incubated overnight at −20 °C. The sample was centrifuged for 15 minutes at 3100 g, 4 °C. Acetone was removed carefully and the pellet was washed 3 times with 1 ml of acetone. The protein pellet was carefully dried at 37 °C and then resuspended in 50 µl of water.

### Derivatization and digestion of histones

Samples were processed by two different proteomic strategies (GeLC−MS/MS and LC−MS/MS) based on the protocol described in our previous study^20^, with some modifications. For GeLC-MS/MS, histone-enriched extracts were resolved on 15% SDS-PAGE gels and stained with Coomassie blue. Histone bands were excised, destained (25 mM NH4HCO3 in 50% ethanol, shaking in a thermomixer at 800 rpm, 25 °C), derivatized with propionylation solution (propionic anhydride in 100 mM NH4HCO3, 1:10)^28^ and digested *in gel*^29^ with trypsin (sequencing modified, Promega) at a concentration of 12.5 ng/uL for 16 h at 37 °C. For LC-MS/MS, histone-enriched extracts were directly derivatized with propionylation solution (propionic anhydride in 2-propanol, 1:3)^30^ and digested *in solution*^30^ with trypsin (sequencing modified, Promega) at a protease/protein ratio of 1/20, for 16 h at 37 °C. For both proteomic strategies, reduction and alkylation were not performed and peptide digests were desalted using C18 StageTips^31^ prior to nanoLC- ESI-MS/MS.

### NanoLC-ESI-MS/MS analysis

Peptide mixtures were separated by online reversed-phase (RP) nanoscale capillary liquid chromatography (nanoLC) and analyzed by electrospray mass spectrometry in tandem (ESI MS/MS). The experiments were performed at the mass spectrometry facility P02-004 (Carlos Chagas Institute - Fiocruz Parana), with an EASY nLC 1000 (Thermo Fisher Scientific) system connected to an LTQ Orbitrap XL (Thermo Fisher Scientific) mass spectrometer equipped with a nanoelectrospray ion source (Phoenix S&T). Chromatographic separation of the peptides took place in a one-column set-up, with a 30-cm analytical column (75 μm inner diameter, 350 μm outer diameter) in-house packed with reversed-phase C18 resin (ReproSil-Pur C18-AQ 1.9 µm, Dr. Maisch GmbH, Ammerbuch-Entringen, Germany), kept at a constant temperature of 60 °C. Solvent A was 0.1% formic acid, 5% DMSO in water, and solvent B was 5% DMSO, 0.1% formic acid in acetonitrile. Samples were injected onto the column and subsequently eluted with a flow rate of 250 nL/min and peptide mixtures were separated with a linear gradient from 5% to 40% acetonitrile in 128 min. The mass spectrometer operated in Data-Dependent Acquisition (DDA) mode to automatically switch between MS and MS/MS (MS^2^) acquisition, using, applying both Collision-Induced Dissociation (CID) and Higher Energy Collisional Dissociation (HCD) to the 5 most intense peptides detected in each MS spectrum. For all samples duplicate or triplicate LC-MS/MS runs were performed. Survey full scan MS spectra (at 300–1600 m/z range) were acquired in the Orbitrap analyzer with resolution R = 60,000 at m/z 400 (after accumulation to a target value of 1,000,000 in the linear ion trap), with preview scan enabled. Singly-charged precursor ions were not selected for fragmentation. Former target ions selected for MS/MS were dynamically excluded for 30 seconds. Total cycle time was approximately three seconds. Other mass spectrometric conditions were: spray voltage, 2.4 kV; no sheath and auxiliary gas flow; ion transfer tube temperature, 100 °C; collision gas pressure, 1.3 mTorr; normalized collision energy using wide-band activation mode 35% for MS2. The ion selection threshold was 250 counts for MS2. An activation q = 0.25 and activation time of 30 ms was applied in MS2 acquisitions. The lock mass^32^ option, using DMSO peaks^33^ was enabled in all full scans to improve the mass accuracy of precursor ions.

### Data analysis

Peptides and hPTM sites were identified with the software Peaks Studio (version 10, Bioinformatics Solutions Inc)^34–36^.The sequential analysis by Peaks Studio started with *de novo* sequencing of fragment spectra (Peptide *De Novo*), followed by peptide sequence match of the high quality de novo tags with (Peaks DB)^36^, considering the most frequent modifications, and then by peptide sequence match of the remaining high quality *de novo* only peptide tags (Peaks PTM)^35^. Proteins were searched against a database containing 20257 sequences of *T. cruzi* Dm28c strain (downloaded on Aug 15, 2018 from TriTrypDB, http://www.tritrypdb.org). In all Peaks searches (Peptide *De Novo*, Peaks DB and Peaks PTM) the precursor mass tolerance was set to 10 ppm and the fragment ion mass tolerance was set to 0.5 Da (ion trap spectra) or 20 ppm (Orbitrap spectra). Minimum peptide size was set to five amino acids, allowing for two missed cleavages. The enzyme for theoretical digestion was Arg-C with specific digestion mode. For Peaks DB, monomethylation (K/R), dimethylation (K/R), trimethylation (K), acetylation (K), acetylation (N-term), propionylation (K), propionylation (N-term), methylpropionylation (K), phosphorylation (S/T/Y) and oxidation (M) were set as variable PTMs. For Peaks PTM, on top of those PTMs searched in PeaksDB, monomethylation (N-term), dimethylation (N-term), glutarylation (K), 2-hydroxyisobutyrylation (K), hydroxylation (Y), malonylation (K), succinylation (K), ubiquitination (K), crotonylation (K), citrullination (R), methylation (Q), phosphorylation (H), butyrylation (K), acetylation (S/T), formylation (K) and deamidation (N/Q) were added as variable PTMs. For identification of both peptides and proteins, the false discovery rate (FDR) was set to 1%. PTM sites with an Ascore ≥20 were automatically validated.

## Data Records

The mass spectrometry proteomics data have been deposited to the ProteomeXchange Consortium via the PRIDE^37^ partner repository with the dataset identifier https://identifiers.org/pride.project:PXD019104^38^. Representative spectra for all novel hPTM marks and additional tables for supporting our dataset have been uploaded to figshare^39^.

## Technical Validation

In the present work, two parallel proteomic approaches (LC-MS/MS and GeLC-MS/MS) were used to deeply profile the hPTMs of canonical, variant and linker histones of *T. cruzi* epimastigotes (Fig. 1). Each proteomic approach was applied to two biological replicates, each of them divided into two or three technical replicates during sample preparation, and multiple LC-MS/MS runs were performed for each sample, totalizing 27.raw files. The experimental design adopted in this study allowed us to substantially expand the repertoire of hPTMs and led to very reliable and complimentary data. One of the reasons for the improved identification of low abundance peptides and more PTM sites was the use of different approaches that not only decreased the complexity of the sample (histone enrichment by acid extraction and further separation by SDS-PAGE), but also explored different biochemical characteristics of histones. The derivatization of proteins before trypsin cleavage prevented overcutting and reduced the charge of the lysine-rich histone regions, especially in the N-terminal tails, producing peptides with good size and charge (doubly and triply charged in electrospray ionization MS^40^) for optimal high energy based peptide identification^41^. After protein propionylation, the samples from both proteomic strategies were directly submitted to the digestion. Thus, this simplified methodology was efficient in the identification of *T. cruzi* hPTMs.

**Fig. 4:**
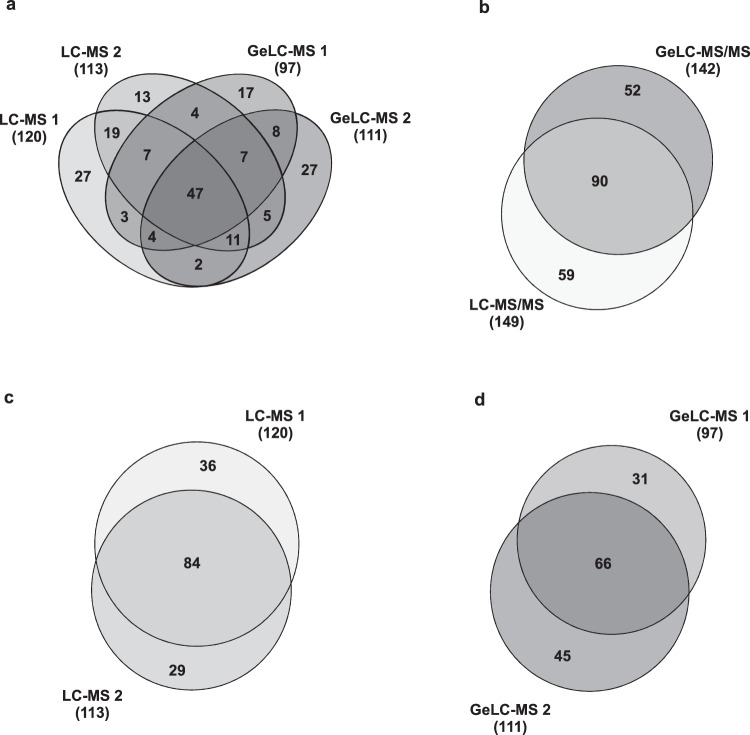
Identification of *T. cruzi* hPTMs across different experiments. Venn diagrams showing the number of hPTMs identified (**a**) each individual experiment, (**b**) different proteomic approaches, (**c**) LC-MS/MS experiments and (**d**) GeLC-MS/MS experiments. The hPTMs contained in each part of the Venn diagrams are listed in figshare File 2^39^.

A combined list of all histone supporting peptides identified in the present work is available in figshare File 1^39^. Each non-redundant peptide sequence was unambiguously identified by multiple features. The identified peptides matched to several distinct gene products that represent each histone in the genome of *T. cruzi*, some of them demonstrating the expression of sequence divergent histone isoforms (Fig. S1a). Among the multiple isoforms detected for each given histone, the one with the highest score and number of PTMs (*e.g*. H2A, BCY84_17381; H2B, BCY84_06298; H3, BCY84_02638; H4, BCY84_15632; H2A.Z, BCY84_22061; H2B.V, BCY84_04421; H3.V, BCY84_18558 and H1, BCY84_14748), was chosen as the model sequence to be used throughout the article. All histones were identified by multiple MS/MS spectra in both biological replicates of the two proteomic strategies (Fig. S1b). The quality of hPTM peptide identification can be verified through their mass accuracy and score distribution (Fig. S2). Also, the majority of hPTMs were detected across multiple samples and experiments, strengthening the reliability of our data (Fig. 4 and figshare File 2^39^).

**Fig. 5:**
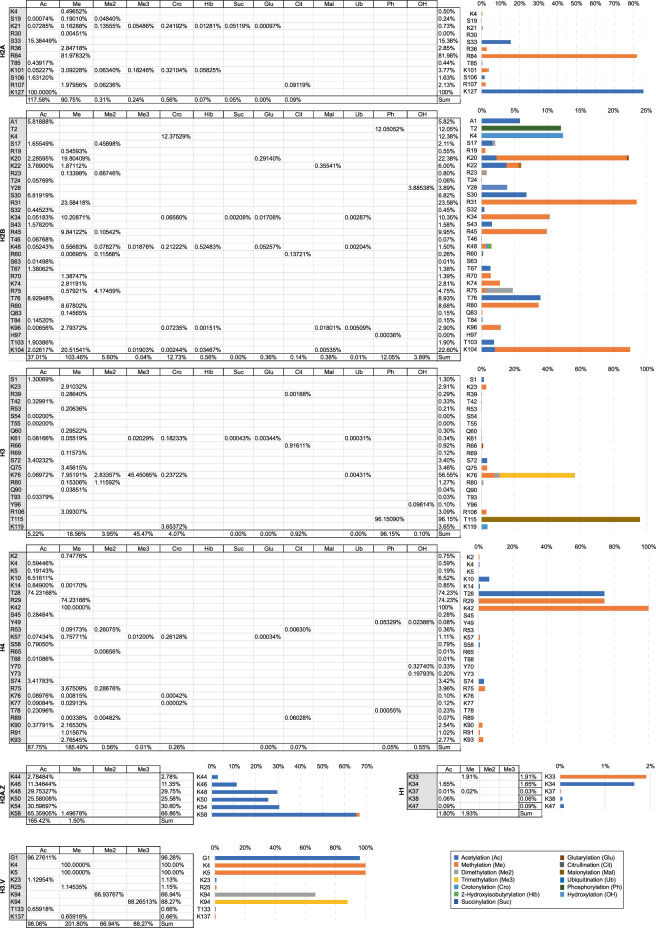
Relative abundance of hPTM sites. For each hPTM site identified the relative abundance (occupancy) were calculated by dividing the intensity of the modified by the sum of the modified and unmodified hPTM site. The cases with 100% of abundance are due to the lack of intensity detection for its unmodified version.

An aspect explored in our data is the relative abundance of hPTMs. In general, the hPTM marks identified displayed low abundance/occupancy. However, some sites of high abundance were also found, mainly for acetylation, methylation and a few phosphorylation sites (Fig. 5). These results seem to be in agreement with previous studies that show the low abundance of scarcely histone modifications in eukaryotes^42^ and that the methylation and acetylation are the most abundant hPTM in *T. cruzi*^19^. Among the hPTM marks with high abundance in our dataset and previously detected in compared studies are the H3K76me/me2/me3, H4K10ac and H4K14ac, important marks involved in cell cycle regulation in trypanosomes^12,14,21^. Other marks in this group are present in variant histones, H3.VK94me2/me3, H2A.ZK54ac and H2A.ZK58ac, that seems to be trypanosome-specific^14^. The relative abundances of the modified peptides and individual modification sites identified in our dataset with a relative abundance equal or higher than 10% are shown in Fig. S3a, b, respectively.

**Fig. 6:**
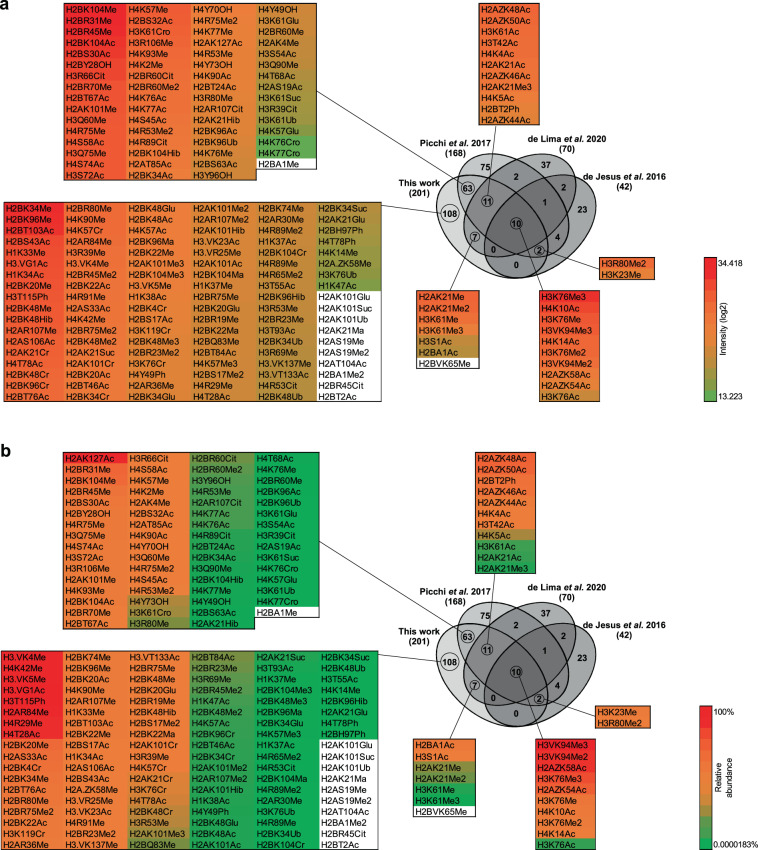
Abundancy of exclusive and confirmed hPTM marks identified in this study compared to previously described analysis. Overlapping hPTMs to other studies are color coded according to (**a**) log2 intensity and (**b**) relative abundance. Detailed data are listed in figshare File 3^39^.

A total of 201 hPTM marks identified in the present work passed the criteria of site localization score (Ascore >20). Among them, 94 confirmed the literature and 107 were newly described in the repertoire of *T. cruzi* hPTMs (representative spectra available in figshare File 5^39^). Also, some hPTMs previously described were not detected here (n = 126) is probably due to technical and biological reasons (e.g. different proteomic strategies, different strains of parasites, regions of histones not covered in a given study, etc.). Also, the chance of identification of hPTM marks in multiple experiments and studies seems to have some correlation with their intensity and relative abundance (Fig. 6 and figshare File 3^39^). In addition to the validated hPTM marks described here, we identified another 111 with Ascore <20, which were not added to our final map. However, several of them were either close to the threshold score and/or identified by multiple features/experiments. Therefore, to allow the reader to fully explore the data, a compiled list of all the hPTM marks identified in the present work (Ascore >20/<20 and literature) are available in figshare File 4^39^.

Our updated and comprehensive profile of *T. cruzi* hPTMs, now available to be used for further studies, reinforces that several residues are targets of multiple modifications, that some modification types are more abundant than others and that hPTMs are widely distributed and diverse at both the tails and the globular domains of all histones, which are both regions with distinct and important roles in the epigenetic regulation of higher eukaryotes^3,43^.

## Supplementary information

Supplementary Figures

## References

[1] Chagas C (1909). Nova tripanozomiaze humana: estudos sobre a morfolojia e o ciclo evolutivo do *Schizotrypanum cruzi* n. gen., n. sp., ajente etiolojico de nova entidade morbida do homem. Mem. Inst. Oswaldo Cruz.

[2] WHO. American Trypanosomiasis (Chagas Disease). *WHO (World Health Organization)* http://www.who.int/en/news-room/fact-sheets/detail/chagas-disease-(american-trypanosomiasis) (2019).

[3] Lawrence M, Daujat S, Schneider R (2016). Lateral thinking: how histone modifications regulate gene expression. Trends Genet..

[4] Arrowsmith CH, Bountra C, Fish PV, Lee K, Schapira M (2012). Epigenetic protein families: a new frontier for drug discovery. Nat. Rev. Drug Discov..

[5] Prachayasittikul V (2017). Exploring the epigenetic drug discovery landscape. Expert Opin. Drug Discov..

[6] Jones, P. A., Issa, J. J. & Baylin, S. Targeting the cancer epigenome for therapy. *Nat. Rev. Genet*. **17** (2016). 10.1038/nrg.2016.93 27629931

[7] Clayton C (2019). Regulation of gene expression in trypanosomatids: living with polycistronic transcription. Open Biol..

[8] Elias MC, Nardelli SC, Schenkman S (2009). Chromatin and nuclear organization in *Trypanosoma cruzi*. Future Microbiol..

[9] Da Cunha JPC (2005). *Trypanosoma cruzi* histone H1 is phosphorylated in a typical cyclin dependent kinase site accordingly to the cell cycle. Mol. Biochem. Parasitol..

[10] Da Cunha JPC, Nakayasu ES, de Almeida IC, Schenkman S (2006). Post-translational modifications of *Trypanosoma cruzi* histone H4. Mol. Biochem. Parasitol..

[11] Respuela P, Ferella M, Rada-Iglesias A, Åslund L (2008). Histone acetylation and methylation at sites initiating divergent polycistronic transcription in *Trypanosoma cruzi*. J. Biol. Chem..

[12] Nardelli SC, Cunha JPC, Motta MCM, Schenkman S (2009). Distinct acetylation of *Trypanosoma cruzi* histone H4 during cell cycle, parasite differentiation, and after DNA damage. Chromosoma.

[13] Marchini, F. K. *et al*. Profiling the *Trypanosoma cruzi* Phosphoproteome. *PLoS One* **6** (2011). 10.1371/journal.pone.0025381 PMC3178638 21966514

[14] De Jesus TCL (2016). Chromatin proteomics reveals variable histone modifications during the life cycle of *Trypanosoma cruzi*. J. Proteome Res..

[15] Amorim JC (2017). Quantitative proteome and phosphoproteome analyses highlight the adherent population during *Trypanosoma cruzi* metacyclogenesis. Sci. Rep..

[16] De Jesus TCL (2017). Quantitative Proteomic Analysis of Replicative and Nonreplicative Forms Reveals Important Insights into Chromatin Biology of *Trypanosoma cruzi*. Mol. Cell. Proteomics.

[17] Moretti NS, Cestari I, Anupama A, Stuart K, Schenkman S (2018). Comparative Proteomic Analysis of Lysine Acetylation in Trypanosomes. J. Proteome Res..

[18] Lucena ACR (2019). Quantitative phosphoproteome and proteome analyses emphasize the influence of phosphorylation events during the nutritional stress of *Trypanosoma cruzi*: the initial moments of *in vitro* metacyclogenesis. Cell Stress Chaperones.

[19] de Lima LP (2020). Improvements on the quantitative analysis of *Trypanosoma cruzi* histone post translational modifications: Study of changes in epigenetic marks through the parasite’s metacyclogenesis and life cycle. J. Proteomics.

[20] Picchi GFA (2017). Post-translational modifications of *Trypanosoma cruzi* canonical and variant histones. J. Proteome Res..

[21] Janzen C, Hake SB, Lowell JE, Cross GAM (2006). Selective Di- or Trimethylation of Histone H3 Lysine 76 by Two DOT1 Homologs Is Important for Cell Cycle Regulation in *Trypanosoma brucei*. Mol. Cell.

[22] Figueiredo LM, Janzen CJ, Cross GAM (2008). A histone methyltransferase modulates antigenic variation in African trypanosomes. PLoS Biol..

[23] Wang QP, Kawahara T, Horn D (2010). Histone deacetylases play distinct roles in telomeric VSG expression site silencing in African trypanosomes. Mol. Microbiol..

[24] Siegel TN (2009). Four histone variants mark the boundaries of polycistronic transcription units in *Trypanosoma brucei*. Genes Dev..

[25] Wright JR, Siegel TN, Cross GAM (2010). Histone H3 trimethylated at lysine 4 is enriched at probable transcription start sites in *Trypanosoma brucei*. Mol. Biochem. Parasitol..

[26] Thomas S, Green A, Sturm NR, Campbell DA, Myler PJ (2009). Histone acetylations mark origins of polycistronic transcription in *Leishmania major*. BMC Genomics.

[27] Camargo EP (1964). Growth and differentiation in *Trypanosoma cruzi*. I. Origin of metacyclic trypanosomes in liquid media. Rev Inst Med trop São Paulo.

[28] Forné, I., Barth, T. & Imhof, A. Quantifying histone modifications using mass spectrometry (Prot 51). *Epigenesys* 1–14 (2012).

[29] Shevchenko A, Tomas H, Havlis J, Olsen JV, Mann M (2006). *In-gel* digestion for mass spectrometric characterization of proteins and proteomes. Nat. Protoc..

[30] Lin S, Garcia BA (2012). Examining Histone Posttranslational Modification Patterns by High Resolution Mass Spectrometry. Methods Enzymol..

[31] Rappsilber J, Mann M, Ishihama Y (2007). Protocol for micro-purification, enrichment, pre-fractionation and storage of peptides for proteomics using StageTips. Nat. Protoc..

[32] Olsen, J. V *et al*. Parts per Million Mass Accuracy on an Orbitrap Mass Spectrometer via Lock Mass Injection into a C-trap. *Mol. Cell. Proteomics* 2010–2021, 10.1074/mcp.T500030-MCP200 (2005). 10.1074/mcp.T500030-MCP200 16249172

[33] Hahne H (2013). DMSO enhances electrospray response, boosting sensitivity of proteomic experiments. Nat. Methods.

[34] Ma B (2003). PEAKS: powerful software for peptide *de novo* sequencing by tandem mass spectrometry. Rapid Commun. Mass Spectrom..

[35] Han X, He L, Xin L, Shan B, Ma B (2011). PeaksPTM: Mass spectrometry-based identification of peptides with unspecified modifications. J. Proteome Res..

[36] Zhang J (2012). PEAKS DB: *De Novo* Sequencing Assisted Database Search for Sensitive and Accurate Peptide Identification. Mol. Cell. Proteomics.

[37] Perez-riverol Y (2019). The PRIDE database and related tools and resources in 2019: improving support for quantification data. Nucleic acid Res..

[38] de Godoy LMF (2021). PRIDE Archive.

[39] de Godoy LMF (2021). figshare.

[40] Garcia BA (2007). Chemical derivatization of histones for facilitated analysis by mass spectrometry. Nat. Protoc..

[41] Frese CK (2011). Improved Peptide Identification by Targeted Fragmentation Using CID, HCD and ETD on an LTQ-Orbitrap Velos. J. Proteome Res..

[42] Simithy J (2017). Characterization of histone acylations links chromatin modifications with metabolism. Nat. Commun..

[43] Tropberger P, Schneider R (2013). Scratching the (lateral) surface of chromatin regulation by histone modifications. Nat. Struct. Mol. Biol..

