# Supplementary material for: An updated map of Trypanosoma cruzi histone post-translational modifications
Source: Sci Data. 2021 Mar 25;8:93. doi: 10.1038/s41597-021-00818-w (PMC7994815; doi:10.1038/s41597-021-00818-w)
Supplement: Supplementary file 1 — Supplementary Figures [file 41597_2021_818_MOESM1_ESM.pdf]

Table of contents

Figure S1. PSM identification of canonical, variant and *T. cruzi* linker histones……...2

Figure S2. Mass accuracy and score distribution of the modified peptides…………...3

Figure S3. The most abundant *T. cruzi* hPTMs in the dataset…………………………4

a

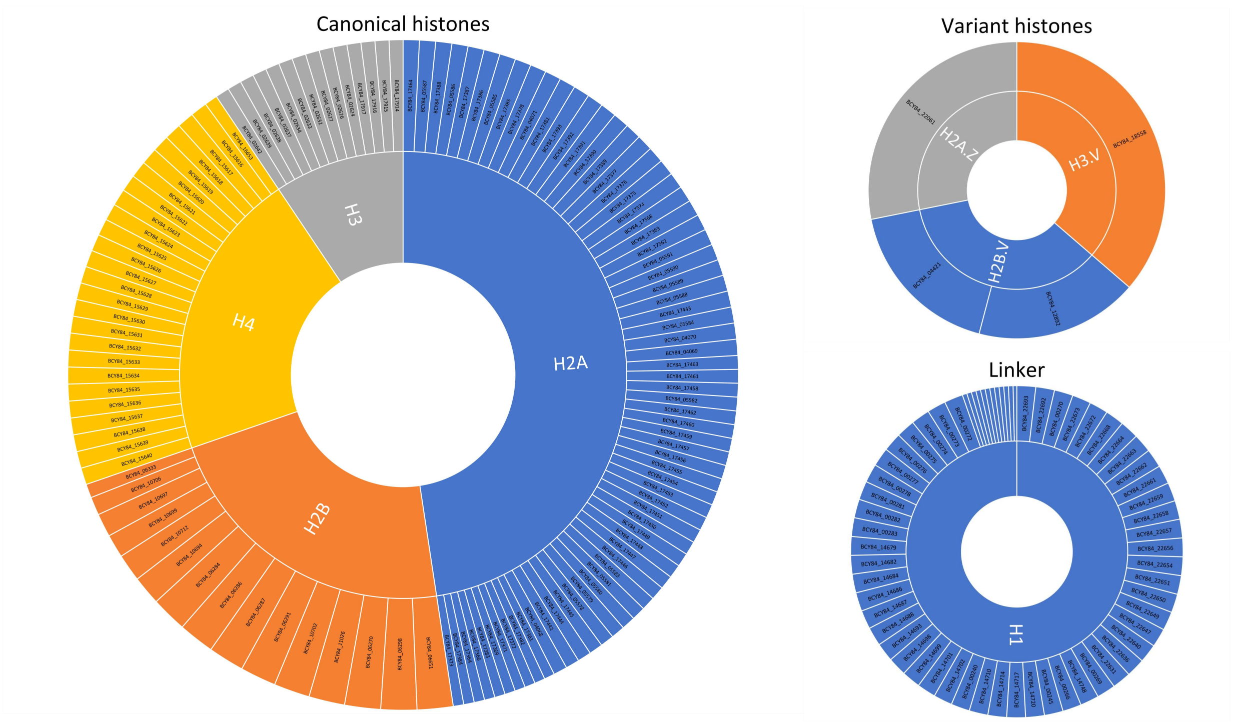

b

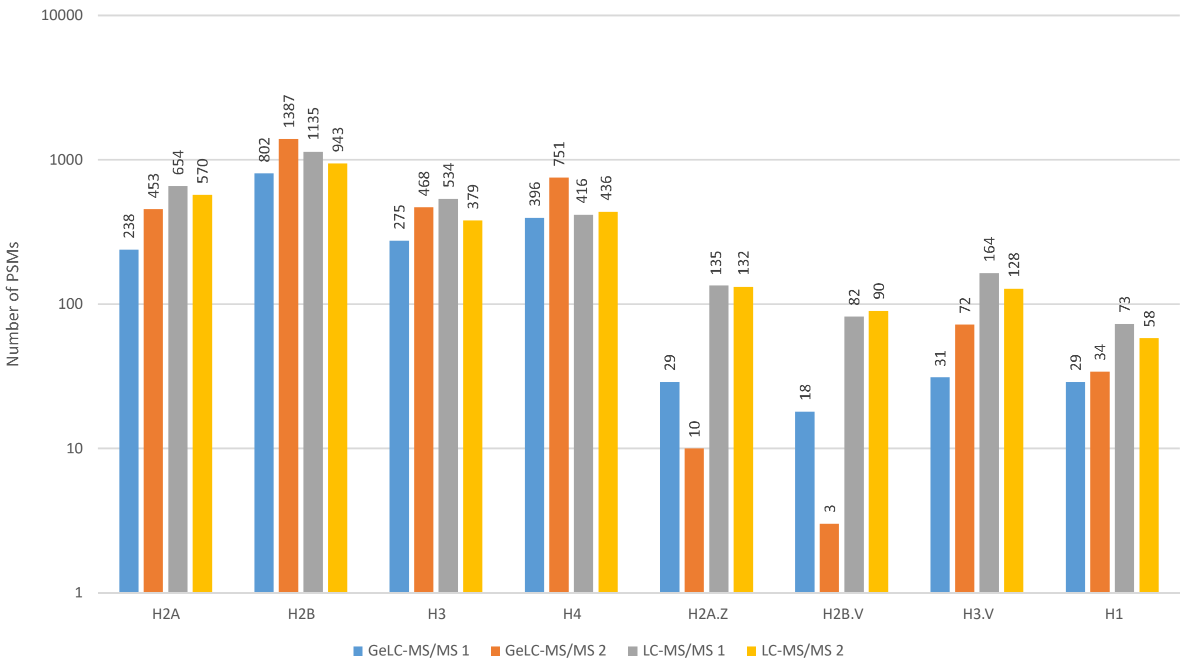

Figure S1. PSM identification of *T. cruzi* canonical, variant and linker histones. (a) Number of PSMs matched to the distinct gene products of each histone. In the outer circle, the angle of each slice is proportional to the number of PSMs identifying that particular sequence. (b) Number of PSMs matched to the supporting peptides for each model histone.

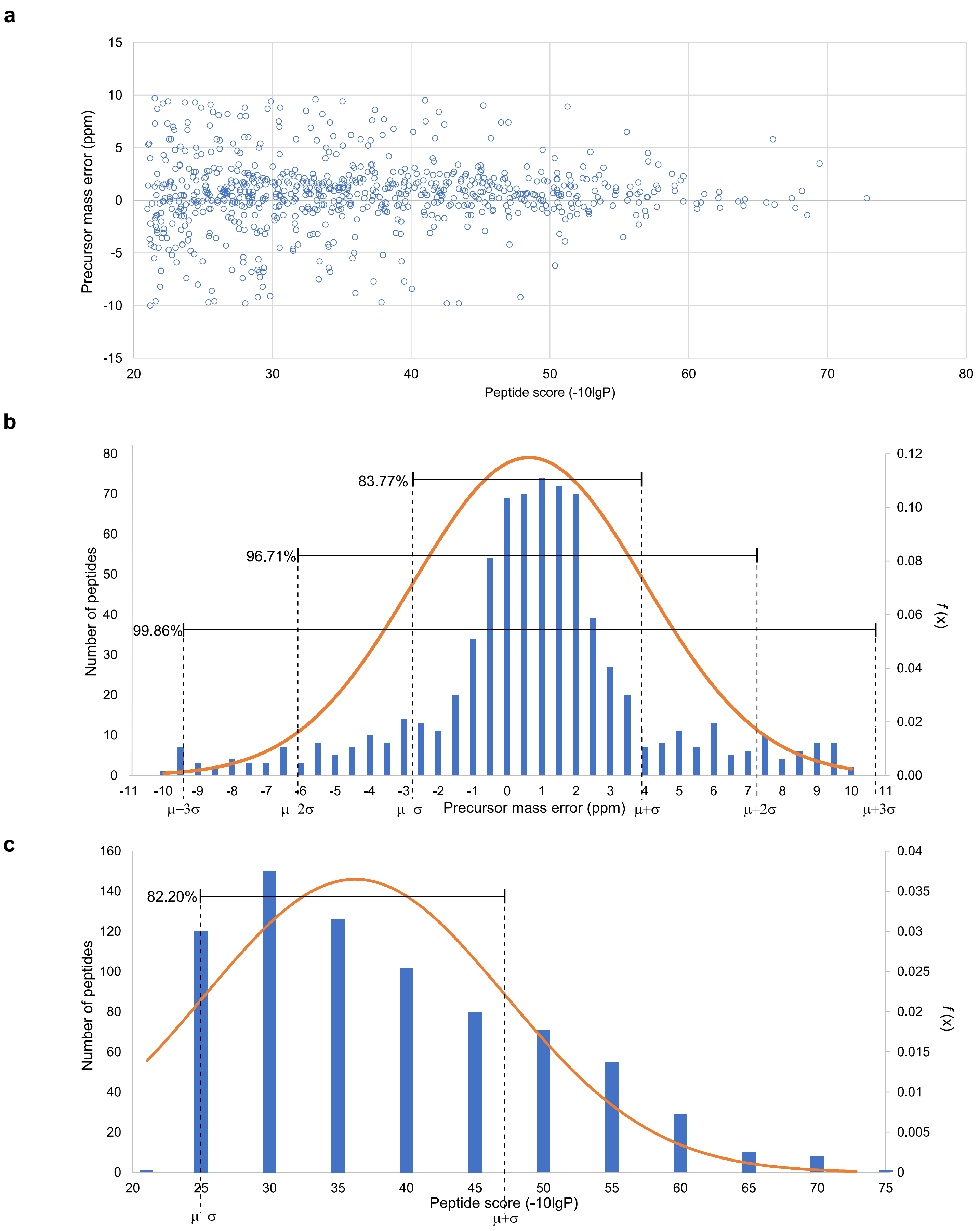

Figure S2. Mass accuracy and score distribution of the modified peptides. (a) Scatterplot of precursor mass error (ppm) versus -10lgP score for modified peptides. (b) Histogram of the precursor mass error distribution of modified peptides. The average mass error of the peptides is +0.63 ppm. (c) Histogram of score distribution of modified peptides, showing that 82.2% of the peptides are between a score range of 25.32 and 47.19.

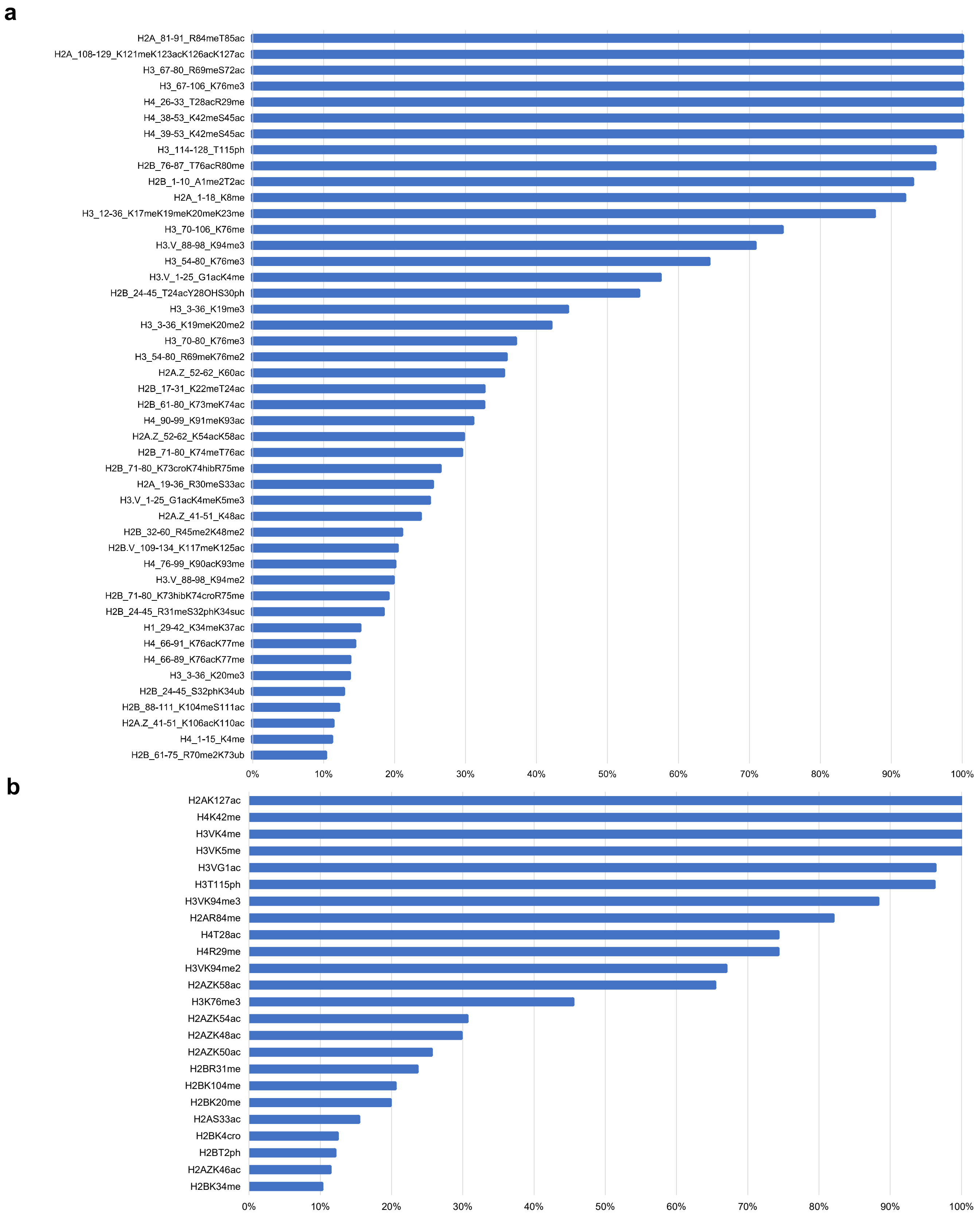

Figure S3. The most abundant *T. cruzi* hPTMs in the dataset. Histone PTM marks with relative abundance above 10% are show for (a) modified peptides and (b) individual sites. The cases with 100% of abundance are due to the not intensity detection of its unmodified version.
